# A mass mimicking pancreatic adenocarcinoma, should hepatobiliary surgeons keep it in mind? a case report

**DOI:** 10.11604/pamj.2021.38.104.25306

**Published:** 2021-02-01

**Authors:** Maria Sotiropoulou, Panagiotis Metaxas, Michail Vailas, Georgios Kyriakopoulos, Paraskevi Alexakou, Michail Psarologos, Charilaos Kyzeridis, Dimitrios Stergiou, Stamatina Koskolou, Stylianos Kapiris

**Affiliations:** 1 Third Department of Surgery, Evangelismos General Hospital, Athens, Greece,; 2 First Department of Surgery, National and Kapodistrian University of Athens, Laikon General Hospital, Athens, Greece,; 3 Department of Pathology, Evangelismos General Hospital, Athens, Greece

**Keywords:** Case report, lung cancer, metastasis, pancreas, surgery

## Abstract

Isolated metastasis to pancreas from lung cancer is an extremely rare entity, usually reported in case series and case reports in the medical literature; estimated to account for up to 3-5% of all pancreatic lesions. Herein, we describe a case of a male patient suffering from metachronous metastatic lesion to the tail of the pancreas secondary to non small cell lung carcinoma treated 4 years prior to his presentation. The patient underwent pancreatic resection due to high clinical suspicion for the malignant nature of the mass, which was proved to be secondary lesion from its prior primary tumor. To the best of our insight this is one of the few reported cases of such type of pancreatic metastasis that may be misleading for hepatobiliary surgeons during preoperative evaluation.

## Introduction

Lung cancer remains the leading cause of cancer-related mortality in Western countries. The most advanced form of this type of malignancy according to tumor-node-metastasis (TNM) classification is stage IV disease, which represents widespread disease with distant metastases [1]. However, the concept of oligometastatic disease (limited number of metastatic lesions in distant organs for which local therapies may be implemented), that has been adopted nowadays in various malignancies, such as colon cancer, could be implemented in order to justify surgical management in lung cancer patients with isolated metastasis and good physical status [2].

Isolated metastasis to pancreas from lung cancer is an extremely rare entity, usually reported in case series and case reports in the medical literature; estimated to account for up to 3-5% of all pancreatic lesions [3]. The most common type of primary tumor that usually metastasizes to the pancreas is reported to be renal cell carcinoma, with the incidence of pancreatic metastases from pulmonary squamous cell carcinoma reaching ~1% of all secondary pancreatic lesions [3, 4]. Many patients remain asymptomatic, whereas common symptoms that may encountered are jaundice and abdominal pain. Interestingly enough, when pancreatic metastasis from lung cancer develops, it is often metachronous and solitary. Thus, differentiation from conventional pancreatic cancer may be difficult and important in the diagnosis [5].

We herein present the case of a male patient suffering from metachronous metastatic lesion to the tail of the pancreas, secondary to non small cell lung carcinoma that was treated 4 years prior to his presentation. The patient underwent pancreatic resection due to high clinical suspicion for the malignant nature of the mass, which was proved to be secondary lesion from its prior primary tumor. To the best of our insight this is one of the few reported cases of such type of pancreatic metastasis that may be misleading for hepatobiliary surgeons during preoperative evaluation.

## Patient and observation

A 51-year old male patient presented to outpatient clinics of our department complaining of abdominal pain and discomfort that was initially presented about two weeks prior to his admission. His history was indicative for surgically treated squamous lung carcinoma 4 years prior to his admission to our hospital. More specifically, he had undergone right pneumonectomy for a stage II squamous cell carcinoma, CK7 (-), p40 (+), CD56 (-), chromogranin (-), synaptophysin (-). After lung resection, patient followed platinum-based chemotherapy regimen and remained disease-free for 4 years.

**Figure 1:**
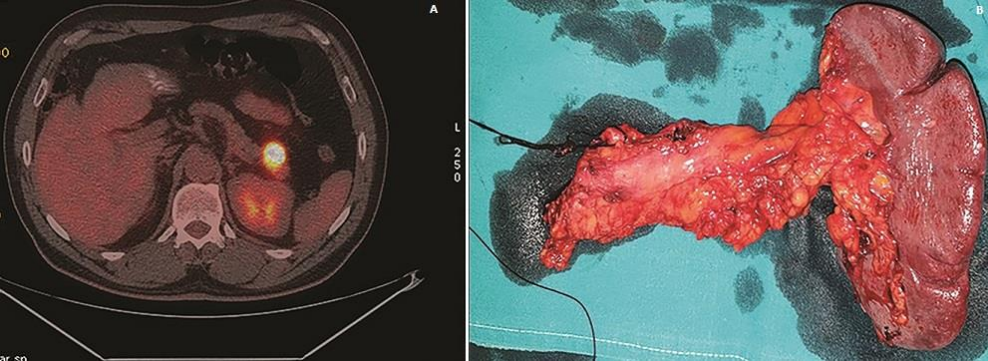
A) PET-CT scan showing hypermetabolic pancreatic tail-mass; B) resected surgical specimen (distal pancreatectomy+splenectomy)

During his admission to our hospital, patient underwent computed tomography (CT) scan which revealed a well-defined mass in the tail of the pancreas approximately 5 mm highly suspicious for malignancy, along with lymphathenopathy. Tumor marker CA 19 9 level was 48.5 U/ml (normal range, 0 37 U/ml). EUS-FNA results were inconclusive and positron emission tomography (PET-CT) confirmed a hypermetabolic mass in the tail of the pancreas, compatible with active malignancy and no distant metastatic disease (Figure 1). After multidisciplinary consultation, surgeon´s decision was to proceed to resection of the mass and informed consent was obtained from the patient. Laparotomy finally was indicative of the aforementioned pancreatic mass with no sign of intra abdominal spread. Distal pancreatectomy and splenectomy were performed (Figure 1).

**Figure 2:**
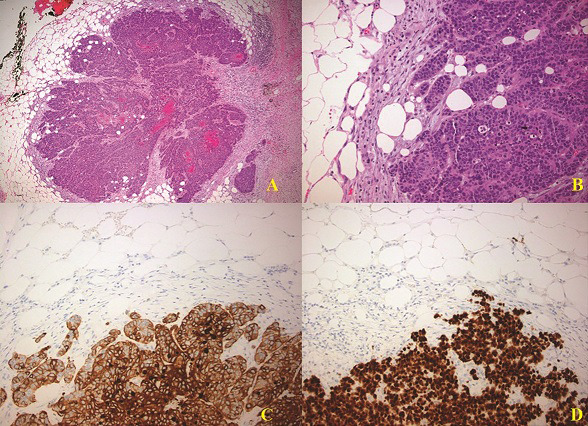
A) keratinized squamous cell carcinoma metastasis in the peripancreatic adipose tissue. HEX40; B) keratinized squamous cell carcinoma metastasis in the peripancreatic fat tissue. HEX200; C) membranous expression of CK5/6 (X200); D) strong and diffuse nuclear expression of p40 (X200)

Histopathological examination revealed two adjacent lesions with an immunohistochemical profile similar to that of the resected primary lung tumor (Figure 2). A single peripancreatic lymph node proved to be positive, showing evidence of the disease. The patient´s postoperative course was uneventful and was discharged seven days after pancreatic resection. He was referred to oncology department and 18 months after pancreatic resection, patient still remains free of disease and secondary metastatic lesions.

## Discussion

Based on the recent medical literature, the most frequent sites of lung cancer metastases are the bones, liver and adrenal glands, with only few case reports reporting isolated pancreatic metastases as an uncommon presentation of this type of malignancy. The incidence of pancreatic metastasis due to squamous cell carcinoma of the lung is reported to be approximately 1.1%, whereas the mean survival time in patients with pancreatic metastasis from lung cancer with-out pancreatectomy is 6.3 months [6].

Oligometastatic metachronous presentation in pancreatic tissue is rare with most of the patients presenting with widespread disease. Therefore, the importance of surgical resection in patients with solitary pancreatic metastasis from lung cancer is inadequately evaluated in the literature [7]. In recent years, efforts have been made to establish treatment guidelines for such patients. Most of the limited data regarding lung cancer oligometastases has focused on aggressive surgical treatment of oligometastases to these sites, with studies reporting considerable difference in overall survival when compared to unresected patients (29 months vs. 8 months) [1].

The clinical presentation of patients with pancreatic metastasis from primary lung cancer varies widely with abdominal pain, weight loss and jaundice frequently reported. In many cases secondary lesions are discovered during the follow-up for their primary malignancy [3]. Clinicians should always keep in mind that pancreatic metastasis may be possible if there is a past medical history of surgically treated lung cancer. However, in many cases differentiation of these lesions from primary pancreatic cancer is quite difficult [8].

A variety of imaging modalities are widely implemented nowadays to assist physicians with the exact diagnosis of a newly discovered pancreatic lesion in such patients. CT scan, PET-CT, endoscopic ultrasound with fine needle aspiration (EUS-FNA) along with tumor markers assessment may play a key role to confirm diagnosis [7]. Interestingly enough, in our case it was not possible to distinguish primary pancreatic tumor from metastatic pancreatic lesions preoperatively. Due to high clinical suspicion, we decided to perform curative resection.

As we mentioned before, metastatic pancreatic lesions from the lung are a rare entity. As a consequence, the effect of surgical resection on the long term survival of such patients is inadequately studied. DeLuzio *et al*. [1] in their review have reported overall median survival for patients undergoing curative intent resection to be 29 months, with 2-year and 5-year survivals of 65% and 21% respectively. On the other hand, palliative surgery or medical only management was associated with a median survival of 8 months and 2-year and 5-year survivals of 25% and 8% respectively [1]. Similarly, Adler *et al*. [9] concluded in their systematic review that radical resection of metastases to the pancreas is feasible and safe, and may confer a survival benefit for selected patients. This recommendation was particularly strong in the case of metastatic renal cell carcinoma [9].

Moreover, Dewamwala *et al*. [10] reported their experience from 13 patients with secondary pancreatic tumors from various non pancreatic primaries, which included renal cell, lung, ovarian, gastric and breast cancer, as well as melanoma (10). The mean postoperative survival following pancreatic resection (8 patients; 61.5%) was 28.9 months (range, 1-91 months) compared with 2 months (range, 5-30 months) for patients who were administered palliative chemotherapy and/or radiation (5 patients; 38.5%) [10]. Pancreatic metastasis from lung squamous cell carcinoma is extremely rare, but as in our case may be successfully managed with surgical resection. However, further studies with large number of patients are required in order to fully elucidate the survival benefit of surgical resection in selected patients with oligometastatic pancreatic disease. Given the fact that patients requiring pancreatic resections after lung resection represent a subset of difficult patient-cases, undergoing a resection by a pancreatic surgeon with a high level of expertise should be considered.

## Conclusion

Curative intent pancreatic resections for oligometastatic lung cancer appear to be associated with an increased survival benefit, when compared to unresected patients according to small studies in the medical literature. Hepatobiliary surgeons should always maintain a high level of clinical suspicion in the management of isolated pancreatic lesions in patients with a history of surgically treated lung cancer.

## References

[1] DeLuzio MR, Moores C, Dhamija A, Wang Z, Cha C, Boffa DJ (2015). Resection of oligometastatic lung cancer to the pancreas may yield a survival benefit in select patients--a systematic review. Pancreatology.

[2] Reyes DK, Pienta KJ (2015). The biology and treatment of oligometastatic cancer. Oncotarget.

[3] Machairas N, Paspala A, Schizas D, Ntomi V, Moris D, Tsilimigras DI (2019). Metastatic squamous cell carcinoma to the pancreas: Report of an extremely rare case. Mol Clin Oncol.

[4] Fujii M, Watanabe K, Kataoka M, Nose S, Shiode J (2015). A case of a pancreatic tumor that was diagnosed as metastasis from lung cancer by endoscopic ultrasound-guided fine needle aspiration. J Med Ultrason (2001).

[5] Reddy S, Wolfgang CL (2009). The role of surgery in the management of isolated metastases to the pancreas. Lancet Oncol.

[6] García Vidal C, Carrillo E, Barreiro B (2003). [Solitary metastasis to the pancreas in a patient with lung cancer]. Arch Bronconeumol.

[7] Kageyama Y, Yamaguchi R, Watanabe S, Aizu K, Sato F, Fujieda H (2019). A long-term survival case after resection of the pancreatic metastasis from lung cancer. Int J Surg Case Rep.

[8] Katsuura Y, Ishida H, Komatsuda T, Kurokawa H (2006). Pancreatic metastasis from lung cancer. J Med Ultrason (2001).

[9] Adler H, Redmond CE, Heneghan HM, Swan N, Maguire D, Traynor O (2014). Pancreatectomy for metastatic disease: a systematic review. Eur J Surg Oncol.

[10] Dewanwala A, Kotowski A, LeVea CM, Ma WW (2012). Secondary Tumors of the Pancreas: Case Report and a Single-Center Experience. J Gastrointest Cancer.

